# *Asymmetron lucayanum*: How many species are valid?

**DOI:** 10.1371/journal.pone.0229119

**Published:** 2020-03-04

**Authors:** Lucie Subirana, Viviana Farstey, Stephanie Bertrand, Hector Escriva

**Affiliations:** 1 Sorbonne Université, CNRS, Biologie Intégrative des Organismes Marins (BIOM), Observatoire Océanologique, Banyuls-sur-Mer, France; 2 The Interuniversity Institute for Marine Sciences, Eilat, Israel; National Cheng Kung University, TAIWAN

## Abstract

The cephalochordates amphioxus or lancelets are benthic marine animals representing the earliest divergent evolutionary lineage within chordates. Although amphioxus are present in most of the world’s tropical and temperate oceans, only about thirty different species grouped into three different genera, *Branchiostoma*, *Epigonichthys and Asymmetron* have been described. In the genus *Asymmetron*, only two species have been characterized, although for one of them, *A*. *lucayanum*, several cryptic lineages exist. In this work we have sequenced and analyzed the mitogenome of an *A*. *lucayanum* population previously described in the Red Sea. The phylogenetic study using this complete mitogenome as well as the analysis of COI gene sequences of several individuals of this Red Sea population show that the Red Sea population is a new cryptic species. We propose to call this new species *Asymmetron rubrum*.

## Introduction

Cephalochordates (i.e. amphioxus) are filter feeding benthic marine animals living in tropical and temperate waters of the oceans worldwide. They are representatives of the most basally divergent group of the chordate phylum, which also includes urochordates and vertebrates [1, 2]. The only detailed publication on the diversity of amphioxus species worldwide in 1996 by Poss and Boschung [3] describes the existence of 50 specific and 10 generic names that have been applied to amphioxus. From these, only 29 represent valid taxa due to the use of numerous synonyms for the same species and genera. The classification of a specimen of amphioxus in one species or another by Poss and Boschung was based on meristic data variation but amphioxus species show a high phenotypic conservation and the morphometric characteristics often overlap between species. Poss and Boschung regrouped these 29 species in only two genera, *Branchiostoma* and *Epigonichthys*, whose major morphological difference is the presence of symmetrical gonads in *Branchiostoma* and asymmetrical dextral gonads in *Epigonichthys*. These authors discussed the possibility of the existence of a third genus, *Asymmetron*, but they could not find any synapomorphy characterizing all species in *Epigonichthys* exclusive of *E*. *lucayanus* (synonym of *Asymmetron lucayanum*). Thus, in the absence of arguments to support the fact that *E*. *lucayanus* (i.e. *A*. *lucayanum*) is the sister-taxon to all other *Epigonichthys*, the authors followed the classification proposed by Richardson and McKenzye [4] with only two genera (*Epigonichthys* and *Branchiostoma*). However, Nohara and colleagues, in 2005 [5], studied the mitochondrial genome of *Epigonichthys* and *Asymmetron* individuals, and they showed that the mitochondrial genome of *Asymmetron* has a different gene order organization compared to those of both *Epigonichthys* and *Branchiostoma*, and, in addition, a phylogenetic study based on 13 mitochondrial genes clearly differentiated three branches among cephalochordates, suggesting the existence of three and not two genera. Thus, today, it is considered that the cephalochordate subphylum contains three genera, two of them showing asymmetrical dextral gonads *(Epigonichthys* and *Asymmetron)* and the third one with symmetrical gonads (*Branchiostoma)*.

The genus *Asymmetron* currently contains only two species, *A*. *lucayanum*, with a circumtropical distribution around the world, and *A*. *inferum*, discovered in 2006 in an anaerobic and sulfide-rich environment caused by a decomposing body of a whale at high depth [6]. However, in 2006, the study of the mitochondrial genome of different specimens of *A*. *lucayanum* from the Atlantic, Pacific and Indian Oceans and the phylogenetic inference obtained from these genomes, clearly showed the existence of three separate branches. This led the authors to propose the presence of three cryptic species [7], one from the West-central Pacific, the second from the Atlantic and the third from the Indo-West Pacific. Nonetheless, a later study published in 2017, using a larger set of mitogenomic data of both *Asymmetron* and *Epigonichthys*, showed that the divergence between the West-central Pacific and the Atlantic clades is low (about 7%) compared to that of these two clades with the Indo-West Pacific clade (about 23%), which has led these authors to propose the existence of only two cryptic species and not three [8].

This study aims to show that the only description of amphioxus species worldwide by Poss and Boschung in 1996 [3] significantly under-evaluated the actual number of species that exist due to the method of classification based on comparative meristic data applied to a phylum with a high phenotypic conservation. For this purpose we have studied a new mitochondrial genome of specimens of a population of *Asymmetron lucayanum* described in the Red Sea in 1962 [9]. We clearly show that specimens from the Red Sea belong to a new clade different from the three previously described within the *Asymmetron* genus. We propose to rename the specimens from the Red Sea as *Asymmetron rubrum*.

## Materials and methods

### Sampling

A total of three adults and eleven planktonic larvae of *A*. *lucayanum* were collected in the Gulf of Aqaba. Adults were collected with a Van Veen grab (300 cm2) at a depth of 5-10m from sandy substratum approximately 100m offshore. The latitude and longitude of the collecting place were, respectively, 29°31'28.6"N and 34°56'09.0"E. Planktonic larvae were collected with a plankton net of 100 μm mesh towed 5 meters below sea surface and with a light-trap equipped with a net of 200μm mesh located at 29°30'06.0"N and 34°55'03.8"E. Collection was performed with a research boat from the Interuniversity Institute for Marine Sciences in Eilat (IUI), Israel. Israel has a law on protected natural values and a permit is only required for those included in it. *Asymmetron lucayanum* is not included so no particular permit is necessary. DNA extraction was undertaken using the CTAB (cetyl-trimethylammonium bromide) method described in B. Winnepenninckx et al (1993) [10].

### Mitogenome sequencing and annotation

Mitochondrial DNA was first amplified by long PCR plus primer walking using a first set of gene-specific primers designed based on the most conserved regions of the *A*. *lucayanum* mitogenome sequences deposited in GenBank (S1 Table). Sequencing of the amplified fragments was performed by Sanger sequencing at the BIO2MAR platform in Banyuls sur Mer, France. The mitogenome annotation of *A*. *lucayanum* from the Red Sea was performed using the mitochondrial genome annotation server MITOS [11] with default parameters and using the invertebrate mitochondrial genetic code.

### Cytochrome c oxidase subunit I sequences phylogenetic analyses

*Cytochrome c oxidase subunit I* (COI) partial nucleotide sequences from the 14 *A*. *lucayanum* specimens collected in the Gulf of Aqaba (Red Sea) were amplified by PCR using the following primers: COI_1R—TGA GGG TGC CCG AAG and COI_1F—TGA CCA GCA ATA GTT. The obtained fragments were subsequently cloned in pGEM^®^-T Easy Vector (Promega) and sequenced at the BIO2MAR platform in Banyuls sur Mer, France. The obtained sequences, together with COI sequences from specimens of the Indo-West Pacific, West-central Pacific and Atlantic clades of *A*. *lucayanum* and from *Branchiostoma belcheri* deposited in GenBank (S1 Table) were aligned using ClustalW[12]. The best model for Maximum Likelihood (ML) analysis was estimated using MEGAX [13]. Distance phylogenetic tree was conducted using BioNJ in Seaview [14] with 1000 bootstrap replicates and using observed distances. ML phylogenetic tree reconstruction was conducted using RAxML-HPC BlackBox v8.2.10 [15] at the CIPRES Portal [16] with 1000 bootstrap replicates. Bayesian Inference (BI) phylogenetic tree was reconstructed using MrBayes v3.2.6 [17] on the CIPRES Portal [16]. Two independent runs were performed, each with 4 chains and one million generations. A burn-in of 25% was used and a consensus tree was calculated for the remaining trees. The resulting tree was customized using FigTree v.1.4.0. A population structure network for each clade was constructed using the program TCS v1.21 [18] with the connection limit fixed at 95%.

### Whole mitochondrial genome analysis

All the protein coding gene sequences from the mitochondrial genomes of a shark (*Scyliorhinus canicula)*, a lamprey (*Petromyzon marinus)*, a hemichordate (*Balanoglossus carnosus)*, and of several cephalochordate species were retrieved from GenBank. The accession numbers are given in S1 Table. Amino acids were used to align all the sequences with the sequences from the mitochondrial genome of *A*. *lucayanum* from the Red Sea using ClustalO [19] implemented in Seaview [14]. All the aligned nucleotide sequences were concatenated. The best model for ML analysis was estimated using MEGAX [13]. Distance phylogenetic tree using observed distances and 1000 bootstrap replicates was reconstructed using BioNJ in Seaview [14]. Maximum Likelihood phylogenetic tree reconstruction was undertaken using RaxML v8.2.10 [15] at the CIPRES Portal [16] under the GTR+I+G model with 1000 bootstrap replications. A BI tree was obtained using MrBayes v3.2.6 [17] on the CIPRES Portal [16]. Two runs with four chains and 500000 generations were set as parameters for the analysis. A burn-in of 25% was used and a consensus tree was calculated for the remaining trees. The resulting tree was customized using FigTree v.1.4.0.

### Divergence time estimation

The timetree was generated using the RelTime method [20] implemented in MEGA X [13] using the BI tree obtained from the whole mitochondrial genome analysis. Divergence times for all branching points were calculated using the Maximum Likelihood method based on the GTR model. Relative times were optimized and converted to absolute divergence times based on two calibration points: the divergence times between vertebrates and cephalochordates (598–787 Ma or 603.55–697 Ma) and between cyclostomes and gnathostomes (524–706 Ma or 412.75–462.50 Ma) [21, 22]. A discrete Gamma distribution was used to model evolutionary rate differences among sites and the rate variation model allowed for some sites to be evolutionarily invariable. The analysis involved 30 nucleotide sequences. All positions containing gaps and missing data were eliminated.

### Nomenclatural acts

The electronic edition of this article conforms to the requirements of the amended International Code of Zoological Nomenclature, and hence the new names contained herein are available under that Code from the electronic edition of this article. This published work and the nomenclatural acts it contains have been registered in ZooBank, the online registration system for the ICZN. The ZooBank LSIDs (Life Science Identifiers) can be resolved and the associated information viewed through any standard web browser by appending the LSID to the prefix “http://zoobank.org/”. The LSID for this publication is: urn:lsid:zoobank.org:pub:7FA60BF1-9D6A-40C9-8EDD-7D5FEB2B3274. The electronic edition of this work was published in a journal with an ISSN, and has been archived and is available from the following digital repositories: PubMed Central and LOCKSS.

## Results

### Genome content and genome organization

**Fig 1:**
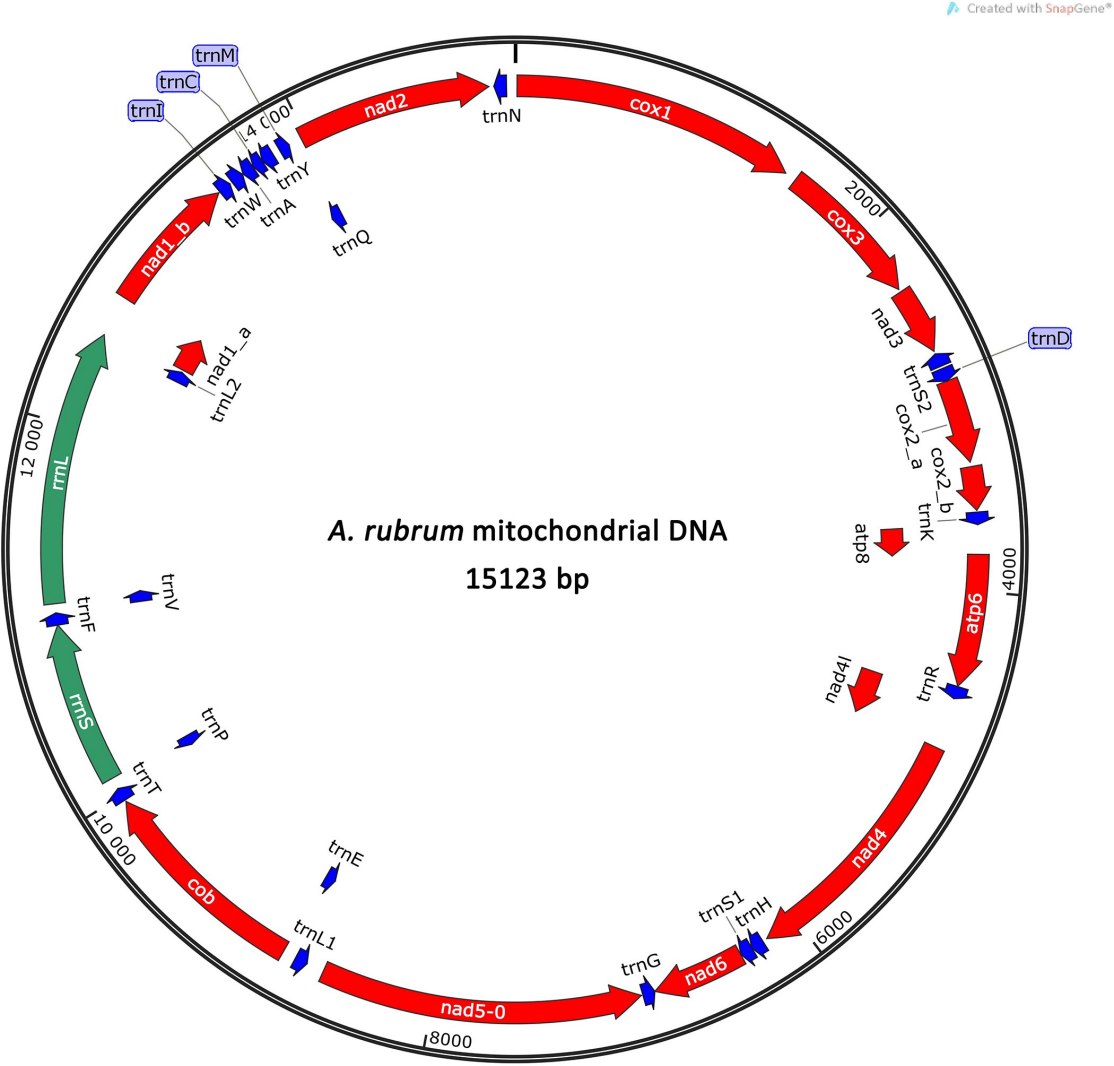
Overview of the mitochondrial genome of *Asymmetron lucayanum* from the Red Sea. Protein coding genes are denoted by three letter abbreviations and labelled in red. Ribosomal genes are denoted by four letter abbreviations and labelled in green. tRNAs are indicated in blue.

The circular mitogenome of *A*. *lucayanum* from the Red Sea is 15123 bp long [GenBank: MN836713] with an AT-content of 60.7% and contains, as other characterized *A*. *lucayanum* mitogenomes, 37 open reading frames, 13 of which code for Oxidative Phosphorylation System proteins (*atp6*, *atp8*, *cox1-3*, *cob*, *nad1-6*, *nad4L*), 22 for tRNAs and 2 for large- and small-subunit ribosomal RNAs (Fig 1 and Table 1). All 13 protein-coding ORFs are transcribed in the same direction except *nad5* which is transcribed in the opposite direction. All protein-coding ORFs start with the canonical translation initiation codon ATG except *cox1* (GTG) and *atp8* (GTG). The 22 tRNAs, which range in size from 55 to 71 nucleotides, are either isolated or clustered in groups of two to seven consecutive genes. The ribosomal genes -*rrnS* and *rrnL-* of *A*. *lucayanum* from the Red Sea are 850 bp and 1441 bp long, respectively. They are located, as in other lancelets, between the *trnP* and *trnL2*(*tta*) genes, being separated by the *trnF* and *trnV* genes.

**Table 1:** Organisation of the *Asymmetron rubrum* mitochondrial genome.

Name	Start	Stop	Strand	Length
cox1	9	1499	+	1491
cox3	1561	2346	+	786
nad3	2366	2716	+	351
trnS2(tca)	2732	2802	-	71
trnD(gac)	2817	2885	+	69
cox2	2886	3575	+	690
trnK(aaa)	3586	3649	+	64
atp8	3650	3820	+	171
atp6	3817	4500	+	684
trnR(cga)	4502	4567	+	66
nad4l	4583	4846	+	264
nad4	4843	6177	+	1335
trnH(cac)	6203	6269	+	67
trnS1(agc)	6270	6335	+	66
nad6	6346	6765	+	420
trnG(gga)	6823	6888	-	66
nad5-0	6896	8587	-	1692
trnL1(cta)	8681	8747	-	67
trnE(gaa)	8757	8820	-	64
cob	8825	9958	+	1134
trnT(aca)	9968	10037	+	70
trnP(cca)	10037	10100	-	64
rrnS	10099	10948	+	850
trnF(ttc)	10949	11011	+	63
trnV(gta)	11012	11078	+	67
rrnL	11061	12501	+	1441
trnL2(tta)	12449	12518	+	70
nad1	12534	13454	+	921
trnI(atc)	13462	13528	+	67
trnW(tga)	13543	13610	+	68
trnA(gca)	13614	13676	-	63
trnC(tgc)	13679	13733	-	55
trnY(tac)	13734	13800	-	67
trnM(atg)	13841	13907	+	67
trnQ(caa)	13907	13975	-	69
nad2	13977	14987	+	1011
trnN(aac)	15014	15079	-	66

The genomic content and the overall arrangement of genes in the Red Sea *A*. *lucayanum* mitochondrial genome is similar to other previously reported mitogenomes of the *A*. *lucayanum* species complex [5–8, 23]. Consequently it differs from *Epigonichthys* and *Branchiostoma* genera in an inversion extending from the *trnL*(*cta*) to the *nad6* gene.

### Phylogenetic distribution and population structure of *Asymmetron* cryptic species

Phylogenetic relationships among *Asymmetron* specimens from different locations were studied using two different datasets: (i) the complete nucleotide sequence of different mitogenomes and (ii) the coding sequences of COI.

**Fig 2:**
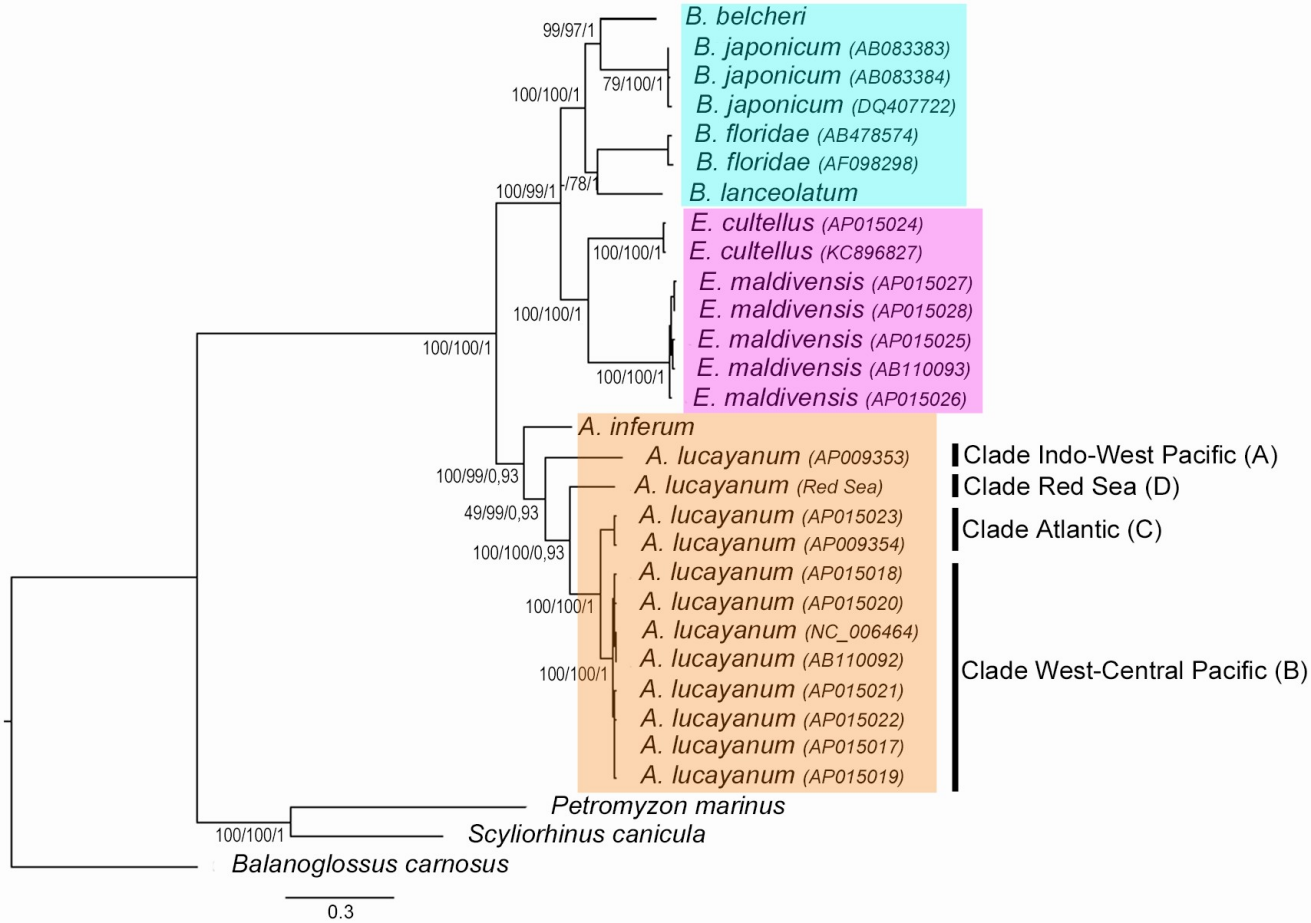
Phylogenetic relationships between cephalochordate species. BI tree for twenty-seven cephalochordates with three outgroups based on whole mitogenome protein coding gene sequences. Numbers on branches refer to bootstraps of the distance tree based on 1000 replicates, bootstraps for the ML analysis based on 1000 replicates and posterior probabilities of the BI tree reconstruction. Scale bar represents the estimated number of nucleotide substitutions per site.

Phylogenetic approaches using Bayesian inference, distance and Maximum Likelihood analyses on the nucleotidic sequence of the complete mitogenome of the Red Sea *A*. *lucayanum*, as well as known mitogenomes of different amphioxus species and lamprey (*P*. *marinus*), shark (*S*. *canicula*) and a hemichordate (*B*. *carnosus*), used as outgroup, allowed the reconstruction of phylogenetic trees showing the same topology (Fig 2). This analysis confirms the results previously reported by other authors [6–8, 24]. Thus, the phylogenetic relationships among the three amphioxus genera show that the *Asymmetron* genus diverged early from the other two genera, *Epigonichthys* and *Branchiostoma*. Concerning the genus *Asymmetron*, we also recover the early divergence of *A*. *inferum* and the presence of a species complex within *A*. *lucayanum* including the Indo-West Pacific (A), West-central Pacific (B) and Atlantic (C) clades [7, 8]. However, the inclusion of the Red Sea *A*. *lucayanum* mitogenome clearly shows the presence of a fourth branch corresponding to a new clade completely independent of the other three within the *A*. *lucayanum* species complex, which we have called Red Sea (D) clade and we have given it the species name *Asymmetron rubrum*, urn:lsid:zoobank.org:act:7CAB510B-377C-490F-8DC8-1FE891D1B14E.

**Fig 3:**
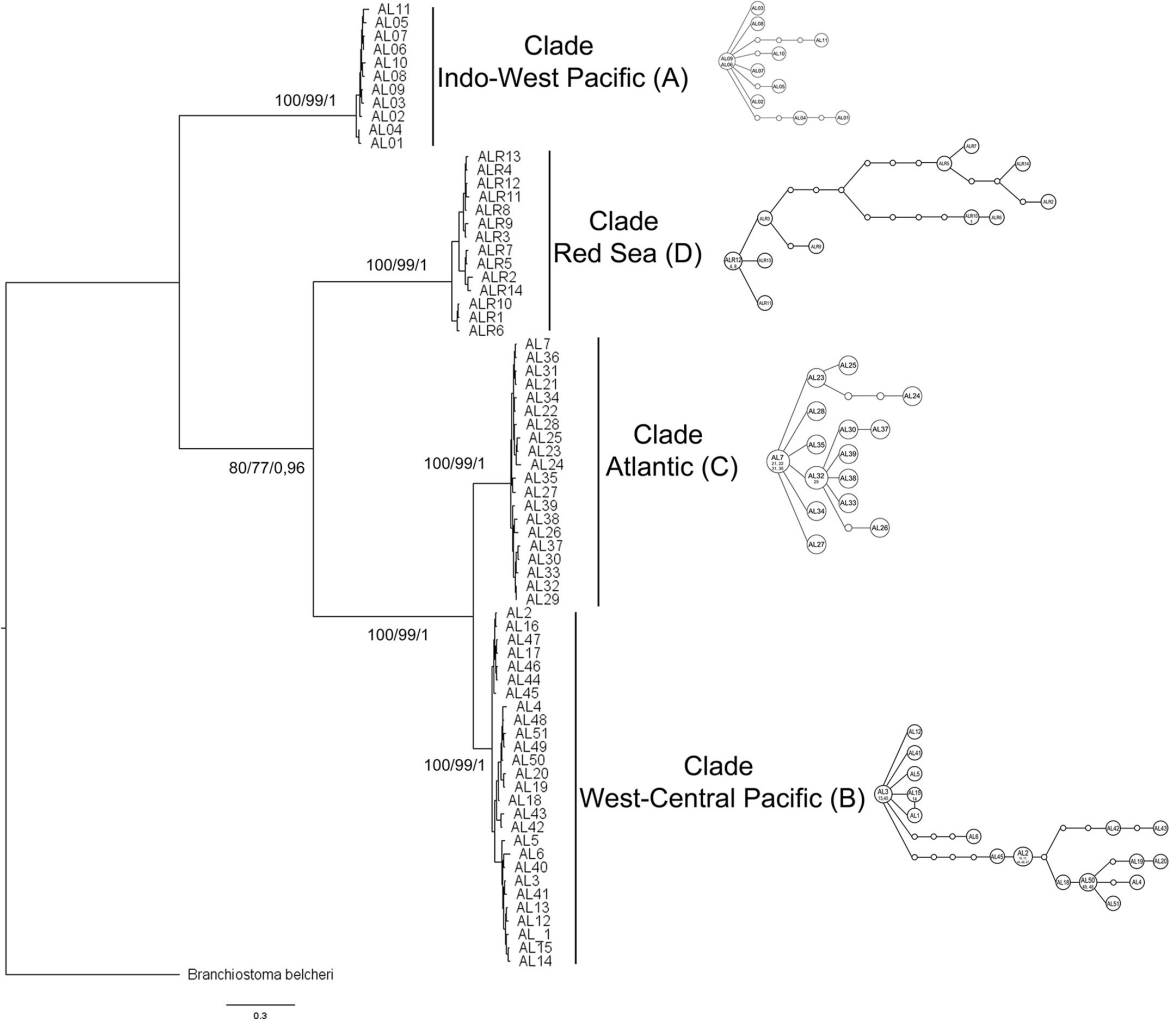
*COI* sequences phylogenetic analysis. Bayesian Inference tree for the *cytochrome c oxidase subunit I* gene for 70 specimens of the lancelet *Asymmetron lucayanum* from the Indo-West Pacific (A), West-central Pacific (B) and Atlantic (C) clades and from the Red Sea (D). *Branchiostoma belcheri* sequence was used as outgroup. Numbers indicate bootstraps based on 1000 replicates for the distance method analysis, bootstrap for the ML analysis based on 1000 replicates, and posterior probabilities of the BI analysis. Scale bar represents the estimated number of nucleotide substitutions per site. On the right side, haplotype networks based on COI sequence alignment for each clade are presented. Sizes of circles indicate haplotype frequency and dots indicate missing haplotypes.

Partial DNA fragments corresponding to the coding sequence of COI (length 650bp) were amplified from 14 specimens collected in the Gulf of Aqaba. The same phylogenetic approaches used for the study of complete mitogenomes were applied to these data with the inclusion in the analysis of COI sequences from different individuals of the three other clades of *A*. *lucayanum* as well as from *B*. *belcheri* (see S1 Table for the accession numbers). The phylogenetic distribution of the different *A*. *lucayanum* specimens using the protein coding sequences of COI confirmed the presence of four clades within the *A*. *lucayanum* species complex that we observed using the complete mitogenome sequences (Fig 3). Eleven different haplotypes could be defined among the 14 specimens of the Red Sea clade of *A*. *lucayanum*. The haplotype network of the Red Sea clade was not connected with the haplotype networks corresponding to the Indo-West Pacific, West-central Pacific and Atlantic clades (Fig 3). Moreover, the genetic distance calculated for the COI genes of the Red Sea (D) clade with the other *A*. *lucayanum* clades (See Table 2), (between 0.170 and 0.203) is comparable to the genetic distances between the Indo-West Pacific and West-central Pacific clades (clades A and B) or the Indo-West Pacific and the Atlantic clades (clades A and C), but higher than the distance observed between the West-central Pacific and the Atlantic clades (clades B and C) (0.053). In addition, this genetic distance is just slightly lower than the genetic distance observed between any of the clades of *A*. *lucayanum* and *B*. *belcheri*.

**Table 2:** Uncorrected p-distance between *Asymmetron lucayanum* clades.

	Clade A	Clade B	Clade C	Clade D
Clade A				
Clade B	0,197			
Clade C	0,202	0,053		
Clade D	0,203	0,172	0,170	
outgroup *B*. *belcheri*	0,228	0,221	0,216	0,222

### Inference of divergence time

**Fig 4:**
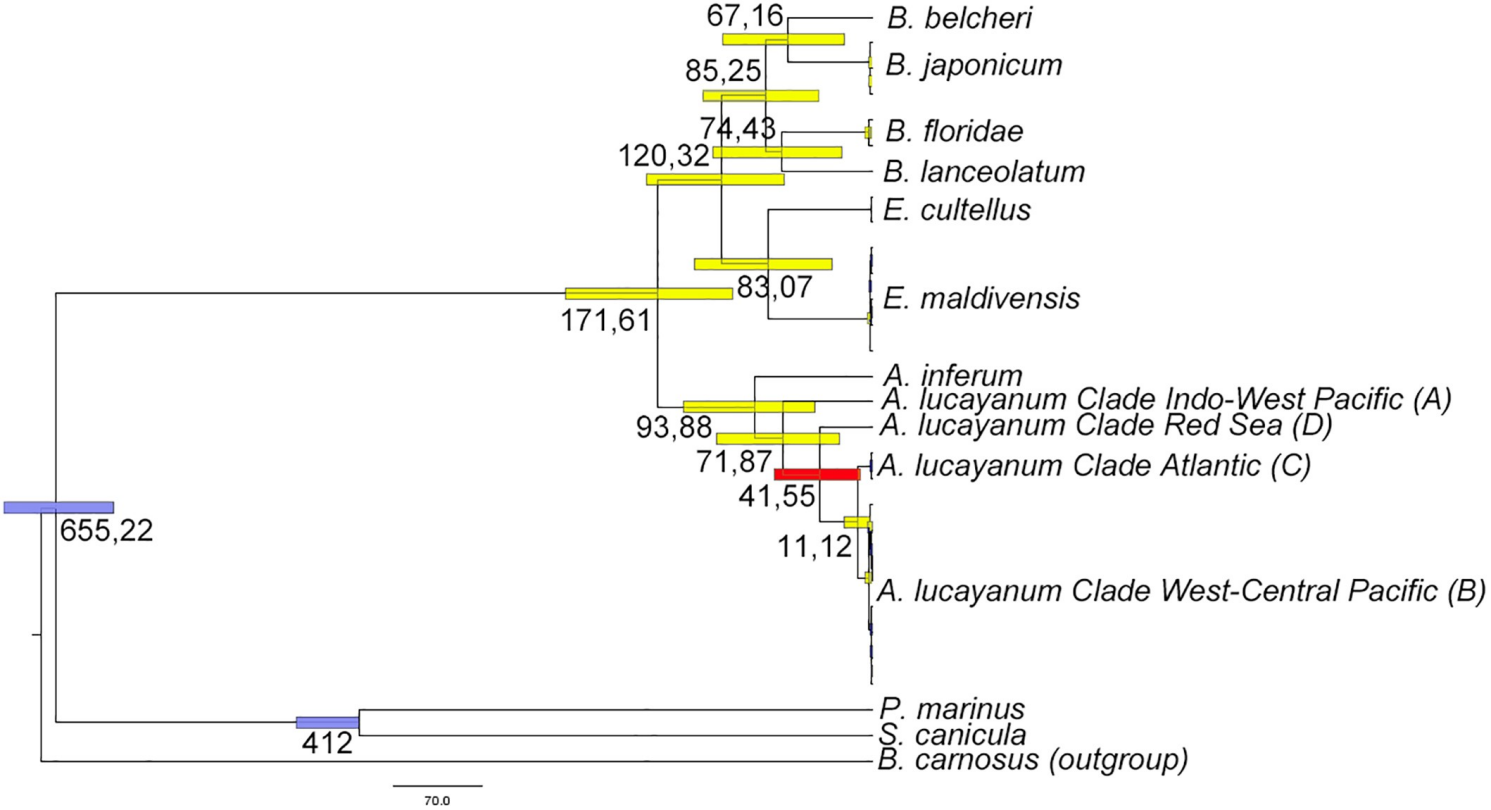
Divergence time estimation between cephalochordates. The timetree was generated using the RelTime method. Two calibration points were used: the divergence times between vertebrates and cephalochordates (603.55–697 Ma) and between cyclostomes and gnathostomes (412.75–462.50 Ma) as proposed by Zhang et al., 2018 [22]. Bars around each node represent 95% confidence intervals. Bars of the calibration points are in blue, bar around the point of divergence of *A*. *lucayanum* from the Red Sea is in red and the others are in yellow. The tree is drawn to scale, with branch lengths measured in the relative number of substitutions per site.

Using the Bayesian inference phylogenetic tree obtained using the different cephalochordate mitochondrial genomes and those of *P*. *marinus*, *S*. *canicula* and *B*. *carnosus*, we have estimated the divergence time between each clade using two different calibrations [21, 22] (Fig 4, S1 Fig and Table 3). Thus, the estimated divergence between cephalochordates and vertebrates occurred around 700 Ma ago (787 Ma or 655 Ma depending on the calibration used, see Table 3). Within the group of cephalochordates, the *Asymmetron* clade is the earliest divergent one and separated from the group *Branchiostoma* + *Epigonichthys* about 200 Ma ago (258.56 Ma or 171.6 Ma depending on the calibration used, see Table 3). Within the group of *Asymmetron* species, *A*. *inferum* is the species that diverged the earliest (152.54 Ma or 93.88 Ma depending on the calibration used, see Table 3), while the radiation of *A*. *lucayanum* in its four cryptic species occurred about ~100 Ma ago between the Indo-West Pacific (A) clade and the other three (116.27 Ma or 71.86 Ma depending on the calibration used, see Table 3), ~50 Ma between the Res Sea (D) clade and the West-central Pacific (B) + Atlantic (C) clades (67.39 Ma or 41.54 Ma depending on the calibration used, see Table 3), and only ~15 Ma between the West-central Pacific (B) and the Atlantic (C) clades (18.03 Ma or 11.12 Ma depending on the calibration used, see Table 3).

**Table 3:** Estimated divergence time for each indicated node.

Node	Hedges et al, 2015 calibration			Zhang et al., 2018, calibration		
	DivTime	CI_Lower	CI_Upper	DivTime	CI_Lower	CI_Upper
Vertebrates/Cephalochordates	787.00	694.66	787.00	655.22	608.74	697.00
Cyclostomes/Gnathostomes	524.00	524.00	615.00	412.00	412.00	462.00
*Asymmetron/Branchiotoma+Epigonichtys*	258.56	212.87	304.26	171.60	111.88	246.08
*Branchiostoma/Epigonichtys*	182.28	144.12	220.45	120.31	70.27	181.21
*E*. *cultellus/E*. *maldivensis*	126.01	87.44	164.59	83.07	32.15	142.54
*B*. *belcheri+B*. *japonicum/B*. *floridae+B*. *lanceolatum*	130.96	98.69	163.22	85.24	42.60	136.04
*B*. *belcheri/B*. *japonicum*	104.27	70.74	137.80	67.15	21.93	119.55
*B*. *floridae/B*. *lanceolatum*	112.34	76.99	147.70	72.43	24.48	128.05
*A*. *inferum/A*. *lucayanum*	152.54	118.35	186.72	93.88	45.51	151.32
*A*. *lucayanum clade A/ A*. *lucayanum clades B+C+D*	116.27	81.98	150.56	71.86	26.34	124.87
*A*. *lucayanum clade D/A*. *lucayanum clade B+C*	67.39	42.47	92.32	41.54	9.46	78.32
*A*. *lucayanum clade C/A*.*lucayanum clade B*	18.03	10.00	26.07	11.12	1.04	22.55

The Estimated divergence time for each indicated node is based on two different calibrations, that of Hedges *et al* ^21^ and that of Zhang *et al* ^22^. Clades A, B C and D correspond to the Indo-West Pacific, West-central Pacific, Atlantic and Red Sea clades, respectively.

## Discussion

The high morphological conservation among cephalochordate species makes it difficult to classify them based on variation of meristic data and such classification probably underestimates the number of species in this subphylum. Indeed, molecular data analyses have revealed that within the genus *Asymmetron* (which contains only two described species, *A*. *lucayanum* and *A*. *inferum*), the species *A*. *lucayanum* actually contains three cryptic species (or at least two, depending on the authors [7, 8]) distributed along the West-central Pacific, the Atlantic and the Indo-West Pacific areas. The phylogenetic tree reconstructions show a close evolutionary relationship between the populations of the West-central Pacific and the Atlantic areas whereas the West-central Pacific and the Indo-West Pacific populations are sympatric in the Pacific area.

In this work we have studied the mitogenome of an amphioxus previously described as *Asymmetron lucayanum* on the Red Sea coasts [9]. The gene content of the mitogenome, as well as the order of the genes, is perfectly conserved with those of the other mitogenomes from individuals of the *Asymmetron* genus that differ from the mitogenomes of the *Branchiostoma* and *Epigonichthys* genera by an inversion of the region extending from the gene *trnL(cta)* to the *nad6* gene. These data suggest that this inversion must have occurred specifically in the ancestor of all *Asymmetron* species approximately 200 Ma ago (between 171 and 258 Ma depending on the calibration used, see Table 3). Our phylogenetic analyses of this mitogenome (either based on the complete mitogenome or on the COI gene) clearly differentiate the Red Sea *A*. *lucayanum* population from the other three previously described populations (see Figs 2 and 3). The divergence time of the Red Sea population is in the Eocene, about 50 Ma ago after the divergence of clade A (Indo-West Pacific, more than 100 Ma) and before the divergence of clades B and C (West-central Pacific and Atlantic, about 15 Ma).

The dispersal routes that could explain the distribution of the different *Asymmetron* populations are subject to controversy since they have been proposed according to two different hypotheses. Both hypotheses can be described as westward hypotheses since the migratory flux is mostly from the Indian Ocean towards the west in both of them. On the one hand it has been suggested that *Asymmetron* originated in the eastern Tethys Sea during the disintegration of Pangea in the Mesozoic. Subsequently, this population expanded towards the east (into the Pacific) and the ancestor of the West Indo-Pacific and Atlantic populations migrated towards the Atlantic, and eventually the Atlantic population expanded into the western Pacific from the Atlantic. The second hypothesis suggests that *Asymmetron* originated in the Indian Ocean and migrated westward, passing through the Neo-Tethys to the Atlantic, and then to the Pacific between North and South America before the closure of the Panama Isthmus. This second hypothesis is based on the absence of evidence of direct eastward migration from the Indian Ocean. The genetic proximity between the Pacific and Atlantic populations suggests that the gene flow between these two groups continued until the formation of the Isthmus of Panama relatively recently (about 3.5 Ma ago). The characterization of the new *Asymmetron* population from the Red Sea, and the inferred geological time at which it originated does not help to establish the migration routes of this species complex. However, the total absence of *Asymmetron* species in the Mediterranean Sea and on the European and African Atlantic coasts allows to propose a third alternative to the two previously enunciated. This third possibility would be an eastward migration from the Indian Ocean, where the genus *Asymmetron* appeared more than 200 Ma ago. Thus, after the origin in the Indian Ocean, giving rise to the Indo-West Pacific population, the ancestor of the Red Sea population migrated towards the North, were the current Red Sea was to form during the Eocene and Oligocene and another population migrated towards the East, to the Pacific Ocean, giving rise to the ancestor of the West-central Pacific and Atlantic clades. Finally, the closure of the Isthmus of Panama separated the Pacific population from the Atlantic population generating the West-central Pacific and Atlantic clades.

The question of species concepts has been and is the subject of extensive literature among biologists. Thus, Mayden, in 1997 [25], listed 20 possible definitions of species, to which more definitions have been added later [26]. The problem is that biologists try to impose a strict classification system on biological processes such as evolution that are continuous and often generate diffuse limits. In other words, whatever concept of species is chosen, we have to admit that the limit will always be arbitrarily imposed by the researcher. In the case of species characterized by low rates of morphological evolution, such as cephalochordates, Highton proposed establishing the limits between species based on the genetic distance between populations (measured as the accumulated number of gene substitutions per locus) [27]. Thus, by comparing genetic distances between well-defined vertebrate species [28], he proposed that populations separated by a genetic distance greater than 0.15 should be considered as different species. In our case, the genetic distance of the *Asymmetron* clades is always greater than 0.15 (see Table 2), except for the distance between clades B and C (West-central Pacific and Atlantic) which is only 0.053. These results suggest that within the four clades of the *Asymmetron* species-complex there are at least three different species. Due to the low genetic distance between the West-central Pacific and the Atlantic clades, we consider that they are the same species as already proposed by Igawa et al [8]. This suggestion of the existence of three different species is supported by the fact that the genetic distance between the two cephalochordate genera, *Asymmetron* and *Branchiostoma*, with clear morphological differences in the symmetry of the gonads, and whose diversification occurred between the Jurassic and the Triassic, about 200 Ma (between 171 and 258 depending on the calibration), is barely higher than that existing between the *Asymmetron* cryptic species (0.17 to 0.20 between *Asymmetron* clades and 0.21 to 0.22 between *Asymmetron* and *Branchiostoma*). Therefore, and keeping this in mind, we consider that the new *Asymmetron* clade from the Red Sea that we describe in this work is a new species and we propose to name it *Asymmetron rubrum*.

## Supporting information

S1 Fig Divergence time estimation between cephalochordates. The timetree was generated using the RelTime method. Two calibration points were used: the divergence times between vertebrates and cephalochordates (598–787 Ma) and between cyclostomes and gnathostomes (524–706 Ma) as proposed by Hedges et al, 2015 [21]. Bars around each node represent 95% confidence intervals. Bars of the calibration points are in blue, bar around the point of divergence of *A*. *lucayanum* from the Red Sea is in red and the others are in yellow. The tree is drawn to scale, with branch lengths measured in the relative number of substitutions per site.
(TIF)

S1 Table GenBank accession numbers for mitogenomes and Cox1 sequences used in this study. (XLSX)
