# Supplementary figures and images for: Asymmetron lucayanum: How many species are valid?
Source: PLoS One. 2020 Mar 4;15(3):e0229119. doi: 10.1371/journal.pone.0229119 (PMC7055842; doi:10.1371/journal.pone.0229119)

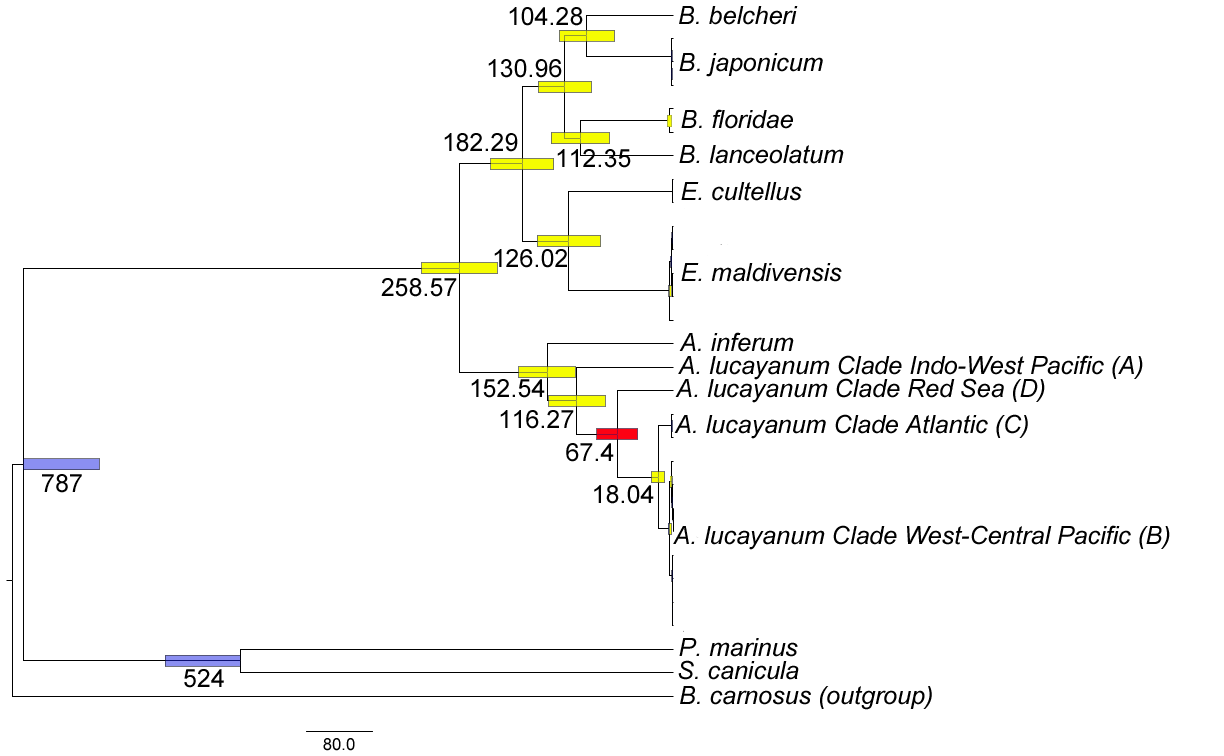

Supplement: S1 Fig — The timetree was generated using the RelTime method. Two calibration points were used: the divergence times between vertebrates and cephalochordates (598–787 Ma) and between cyclostomes and gnathostomes (524–706 Ma) as proposed by Hedges et al, 2015 [21]. Bars around each node represent 95% confidence intervals. Bars of the calibration points are in blue, bar around the point of divergence of A. lucayanum from the Red Sea is in red and the others are in yellow. The tree is drawn to scale, with branch lengths measured in the relative number of substitutions per site. (TIF) [file pone.0229119.s001.tif]
